# Tapped area detection and new tapping line location for natural rubber trees based on improved mask region convolutional neural network

**DOI:** 10.3389/fpls.2022.1038000

**Published:** 2023-01-10

**Authors:** Yaya Chen, Heng Zhang, Junxiao Liu, Zhifu Zhang, Xirui Zhang

**Affiliations:** ^1^ School of Information and Communication Engineering, Hainan University, Haikou, China; ^2^ Mechanical and Electrical Engineering College, Hainan University, Haikou, China

**Keywords:** Mask RCNN, object detection, image segmentation, attention mechanism, natural rubber tree, new tapping line location

## Abstract

Aiming at the problem that the rubber tapping robot finds it difficult to accurately detect the tapped area and locate the new tapping line for natural rubber trees due to the influence of the rubber plantation environment during the rubber tapping operation, this study proposes a method for detecting the tapped area and locating the new tapping line for natural rubber trees based on the improved mask region convolutional neural network (Mask RCNN). First, Mask RCNN was improved by fusing the attention mechanism into the ResNeXt, modifying the anchor box parameters, and adding a tiny fully connected layer branch into the mask branch to realize the detection and rough segmentation of the tapped area. Then, the fine segmentation of the existing tapping line was realized by combining edge detection and logic operation. Finally, the existing tapping line was moved down a certain distance along the center line direction of the left and right edge lines of the tapped area to obtain the new tapping line. The tapped area detection results of 560 test images showed that the detection accuracy, segmentation accuracy, detection average precision, segmentation average precision, and intersection over union values of the improved Mask RCNN were 98.23%, 99.52%, 99.6%, 99.78%, and 93.71%, respectively. Compared with other state-of-the-art approaches, the improved Mask RCNN had better detection and segmentation performance, which could better detect and segment the tapped area of natural rubber trees under different shooting conditions. The location results of 560 new tapping lines under different shooting conditions showed that the average location success rate of new tapping lines was 90% and the average location time was 0.189 s. The average values of the location errors in the *x* and *y* directions were 3 and 2.8 pixels, respectively, and the average value of the total location error was 4.5 pixels. This research not only provides a location method for the new tapping line for the rubber tapping robot but also provides theoretical support for the realization of rubber tapping mechanization and automation.

## 1 Introduction

Natural rubber is an indispensable industrial raw material and a strategic material. Tapping rubber trees is the most important way to obtain natural rubber (LimaGouvêa et al., 2022; Qin et al., 2022). Rubber tapping is the central link and key technical link of rubber production, which requires high physical and technical requirements of laborers, and its labor input accounts for about 70% of the entire natural rubber production (Meksawi et al., 2012; Zhang et al., 2019). At present, natural rubber tapping mainly uses manual tapping, and the commonly used tapping tools include traditional tapping knives, hand-held electric tapping knives, etc. (Arjun et al., 2016; Soumya et al., 2016; Zhou et al., 2021)

Manual rubber tapping is labor-intensive, time-consuming, and laborious, with low work efficiency and high labor costs (Zhang et al., 2022a). Therefore, there is an urgent need for an automatic machine with a simple structure, high stability, and suitability for natural rubber tapping to realize the natural rubber tapping operation. In order to realize the mechanization and automation of natural rubber tapping, it is particularly important to detect the tapped area and locate the new tapping line for natural rubber trees. In the rubber tapping operation of natural rubber trees, the environment of the rubber plantation is complex (uneven light, similar colors of the object and the environment, etc.), and the appearance (color, texture, shape, etc.) of the tapped area of natural rubber trees of different varieties, tree ages, and tree shapes is greatly different. These unstructured and uncertain factors make it difficult to detect the tapped area and locate the new tapping line for natural rubber trees.

In recent years, with the development of computer vision technology, machine vision has been widely used in the field of agricultural engineering (Rehman et al., 2019). Some scholars have researched the detection technology of the natural rubber tree tapping line and have achieved some results. Wongtanawijit and Khaorapapong (2022) used image differencing with a connected component labeling algorithm and the sub-array searching technique to detect the natural rubber tree tapping line under low light conditions. Sun et al. (2022) detected the natural rubber tree tapping line based on the threshold segmentation, binary processing, morphological operation, and edge extraction operator of traditional machine vision. The traditional computer vision processing method is adopted in the above method. The type of tapping line detected by the method is single, and the adaptability to situations such as no rubber liquid flowing out of the natural rubber tree, a complex rubber garden environment, and large light changes is poor. Compared with the traditional methods above, the object detection algorithms in deep learning have a strong feature extraction ability and self-learning ability, which are widely used in crop object detection (Coulibaly et al., 2022). Wan and Goudos (2020) adopted the improved Faster RCNN to realize the detection of multiple types of fruits. Song et al. (2019) used VGG16 to construct and train a Faster RCNN model to detect kiwifruit under different lighting conditions. Bai et al. (2022) proposed an automatic cucumber segmentation and recognition method combining data processing, single-stage object recognition networks (YOLO-v3 and SSD), the U-Net semantic segmentation network, and migration learning to improve the accuracy of localization and grasping ability of cucumber-picking robots. Zhang et al. (2022b) proposed an RTSD-Net network based on YOLOv4-tiny for real-time detection of strawberries in the indoor environment. However, the abovementioned deep neural network algorithms (such as Faster RCNN, YOLO, and SSD) can only roughly calculate the object position through the bounding box and can't accurately obtain the object profile and shape information (Yu et al., 2019). As we all know, similar to circular targets such as apples and kiwifruit, the location accuracy of the bounding box can already meet the location requirements of the object (Liu et al., 2019). In contrast, rubber tapping can only be carried out along the existing rubber tapping line of natural rubber trees to prevent tree damage and production reduction. Therefore, the location of the new tapping line of natural rubber trees requires a high precision profile and shape recognition of the tapped area, which means that the above method can't meet the detection requirements of the tapped area of natural rubber trees, and pixel-level segmentation with higher accuracy is required. Currently, pixel-level segmentation methods based on deep learning include FCN, SegNet, DeepLab, Mask RCNN, etc. (Jia et al., 2020; Peng et al., 2020; Wang et al., 2021) Among them, FCN, SegNet, and DeepLab can only achieve semantic segmentation tasks (Badrinarayanan et al., 2017; Shelhamer et al., 2017; Yurtkulu et al., 2019). While Mask RCNN (He et al., 2017) integrates the object detection task and the semantic segmentation task into a single framework by adding an FCN (Shelhamer et al., 2017) branch to the back end of the Faster RCNN (Ren et al., 2017) framework. In this way, Mask RCNN has both the functions of object detection and semantic segmentation, which greatly improves the accuracy of object detection. In an unstructured environment, Mask RCNN can not only obtain a high accuracy of target detection on the tapped area of natural rubber trees but also obtain the mask of target pixel-level segmentation in the image to be detected. Moreover, Mask RCNN is very flexible and can be used to complete a variety of tasks, including object classification, object detection, semantic segmentation, instance segmentation, and other tasks, which improves the generalization ability of the detection algorithm (He et al., 2017). In addition, the research on the application of Mask RCNN related technology to detect the tapped area and locate the new tapping line for natural rubber trees has not been reported. To sum up, this paper uses Mask RCNN to detect the tapped area and locate the new tapping line for natural rubber trees. However, the original Mask RCNN framework is designed to meet the detection needs of thousands of different types of objects (Liu et al., 2019). It is difficult to achieve its best effect when it is only used for the detection of the tapped area and the location of the new tapping line for natural rubber trees. There are some shortcomings, such as the poor ability to extract subtle features in natural rubber tree images, the low segmentation accuracy of the detail part, the poor network detection accuracy and segmentation accuracy, and the poor quality of the generated mask effect. To this end, this paper proposes a method for detecting the tapped area of natural rubber trees and locating the new tapping line based on the improved Mask RCNN to realize the mechanization and automation of natural rubber tapping operations. The main innovations and contributions are summarized as follows:

(1) The dataset of natural rubber tree tapped area detection and new tapping line location was established. The image was preprocessed by using bilinear interpolation, data enhancement, and other methods to diversify the image dataset, enhance the anti-interference ability under complex conditions, and improve the network training model effect and generalization ability.
(2) The attention mechanism was fused into the ResNeXt to enhance the feature extraction capability of the backbone network, the relevant parameters of generating the anchor box were modified in the region proposal network to improve the matching degree between the anchor box and the natural rubber tapped area, and a tiny fully connected layer branch was added into the mask branch to improve the mask quality to improve the Mask RCNN. The improved Mask RCNN was used to realize the detection and rough segmentation of the tapped area of natural rubber trees.
(3) On the basis of the detection and rough segmentation of the tapped area, the fine segmentation of the existing tapping line of natural rubber trees was realized by combining the edge detection and logic operation. Then, the existing tapping line was moved down a certain distance along the center line direction of the left and right edge lines of the tapped area to obtain the position of the new tapping line, and the new tapping line was smoothed to realize the location of the new tapping line.
(4) The tapped area detection and the new tapping line location method based on improved Mask RCNN were trained and tested to accurately detect and segment the tapped area and effectively locate the new tapping line.

The method proposed in this paper not only provides technical support for the location of the new tapping line of the rubber tapping robot in the rubber garden environment but also provides theoretical support for the realization of mechanization and automation of rubber tapping. The rest of this paper is organized as follows: The "Materials and Methods" section introduces the dataset and methods adopted in this study. In the "Results and Discussion" section, the experimental results of the performance evaluation of the natural rubber tree tapped area detection model and the experimental results of the new tapping line location are presented, and the experimental results are discussed. Finally, the "Conclusion and Future Work" section gives the main conclusions of the study and makes suggestions for future research.

## 2 Materials and methods

### 2.1 Image acquisition

**Figure 1:**
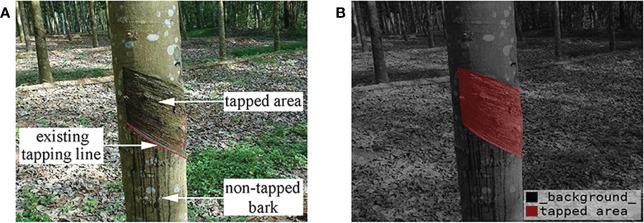
Natural rubber tree images. **(A)** Acquired natural rubber tree image and **(B)** visualization of mask image.

In this research, natural rubber trees in their natural growth state were taken as the test objects, and a Sony DSC-RX100 camera was used for multi-angle shooting. The imaging range was 400-800 mm, and the image resolution was 5472×3648 pixels. To ensure the diversity of image samples, the dataset was divided into direct sunlight on sunny days, backlight on sunny days, and cloudy days according to the light conditions at the time of the shooting, and was divided into one year, two years, and three years according to the year when the natural rubber tree tapped area had been cut at the time of the shooting. A total of 1800 images of natural rubber trees were collected in the natural rubber garden in Danzhou, Hainan, China. All images in the dataset included the tapped area, the existing tapping line (the existing tapping line was on the tapped area) (**Figure 1A**), the natural rubber tree, and the complex rubber garden environment.

### 2.2 Image preprocessing

To reduce the training time of the network model, a bilinear interpolation algorithm (Du et al., 2022) was used to scale the acquired images to 652×552 pixels. To improve the effectiveness of the network training model and the generalization ability of the model, the data enhancement method was adopted to increase the number of natural rubber tree image samples for the collected images and prevent the network from being over-fit due to insufficient training samples. The dataset images were expanded to 3600 by a random combination of increasing and decreasing brightness, color, contrast ratio, and Gaussian noise. After the image preprocessing, the data labeling software Labelme was used to manually mark the polygons of the image to complete the production of the image data label. During labeling, only the tapped area (**Figure 1B**) in the natural rubber tree image was marked. 560 images of the whole dataset were randomly selected as the test set, and the remaining 3040 images were selected as the training set.

### 2.3 Improved Mask RCNN's natural rubber tree tapped area detection and segmentation model

**Figure 2:**
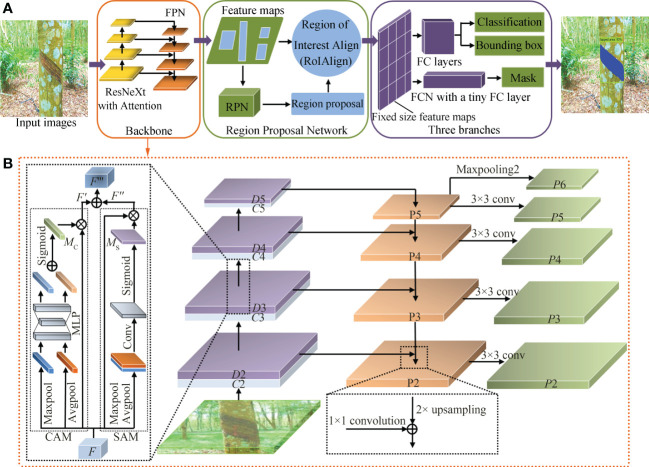
**(A)** Structure of natural rubber tree tapped area detection and segmentation method based on improved Mask RCNN and **(B)** improved backbone network structure.

The convolutional neural network algorithm based on region is the most representative method in the current object detection field. As a relatively new achievement in this series, Mask RCNN has a very flexible framework, which can add different branches to complete different tasks and can complete object classification, object detection, semantic segmentation, instance segmentation, human posture recognition, and other tasks (He et al., 2017). In this paper, the improved Mask RCNN object detection model is used to identify, detect, and segment the tapped area of natural rubber trees, mainly to improve and optimize the backbone network, structural parameters, and the mask branch of the Mask RCNN. **Figure 2A** shows the structure of the natural rubber tree tapped area detection and segmentation method based on the improved Mask RCNN network. First, input images went through the backbone network composed of a ResNeXt fused with the attention mechanism and a feature pyramid network (FPN) for feature extraction. Then, feature maps were input into the region proposal network (RPN) to generate the region proposals. Region of interest align (RoIAlign) extracted features from each region proposal and aligned them one-to-one with the input of the RPN to generate fixed size feature maps. Finally, two parallel operations were performed. Classification and bounding box regression of the tapped area were achieved by the fully connected (FC) layers, and a high-accuracy segmentation mask was generated by the FCN with a tiny FC layer to obtain the area where the tapped area was located. The details will be elaborated on in the following subsections.

#### 2.3.1 Backbone network

The backbone network is a feature extraction network, which extracts features from images to facilitate subsequent image processing tasks (Wang and He, 2022). The original Mask RCNN network uses ResNet and FPN to form the backbone network for feature extraction, which has some problems. When the ResNet is deep or has many parameters, issues such as poor subtle feature extraction ability, low segmentation accuracy of the detail part, and gradient dispersion are common. To solve the above problems, improve the accuracy of the model in extracting the features of the tapped area, prevent the model from generating gradient dispersion, and reduce the use of hyper-parameters, the improved Mask RCNN adopted a ResNeXt fused with an attention mechanism and FPN to form the backbone network to extract the features of the tapped area. As shown in **Figure 2B**, the parallel connected attention mechanism module, the convolutional block attention module (CBAM), was integrated at the end of each level from *C*2 to *C*5 of the ResNeXt to enhance the subtle feature extraction ability of the backbone network and improve the network detection and segmentation accuracy.

(1) ResNeXt

**Figure 3:**
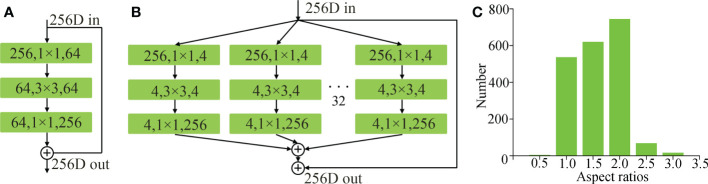
**(A)** A block of ResNet, **(B)** a block of ResNeXt, and **(C)** aspect ratio distribution of tapped area.


The block of the ResNeXt (Panta et al., 2020) (shown in **Figure 3B**) combines the residual block of the ResNet (shown in **Figure 3A**) and the structural characteristics of the split-transform-merge of the inception network, selects a consistent topological structure to realize hyper-parametric sharing, and changes the number of branches through the number of groups. The block of ResNeXt greatly improves the scalability of the model and improves the accuracy of network detection without increasing the complexity of parameters. The split-transform-merge structure of the ResNeXt is expressed by formula (1).

(1) $$F_{y}=F_{x}+\sum_{i=1}^{c}T_{i}(F_{x})$$

Where *F_x_* is the input feature, *F_y_* is the output feature, *T_i_* is the same branch structure, and *c* is the number of branches, that is, cardinality. In this model, *c* is 32.

(2) Attention mechanism

The attention mechanism adopted in deep learning is similar to the selective visual attention mechanism of human beings. The attention mechanism enables the model to select the information most critical to the current task from a large amount of information during the training process. The attention mechanism allows the neural network to focus on the relevant elements of the object in the input image while suppressing the irrelevant elements (Wang and He, 2022). As a lightweight attention mechanism module, CBAM (Ma et al., 2022) is composed of two parts: the channel attention module (CAM) and the spatial attention module (SAM). CBAM pays attention to features of the channel and space, which not only saves parameters and computational power but also ensures that it can be integrated into the existing network architecture as a plug-and-play module. Since CBAM is a serial structure, CAM has a certain degree of influence on the features learned by SAM. Therefore, in this study, CBAM adopted a parallel connection method to carry out feature fusion on the outputs of the two modules by element, so that there was no need to pay attention to the ordering of SAM and CAM.

Let the output *F*∈*R^C^* ^×^ *^H^* ^×^ *^W^* of layer *C*3 be the input feature map of CBAM, as shown in **Figure 2B**. First, *F* passed the global max-pooling and global average-pooling in parallel to obtain two feature maps with a size of *C*×1×1 and two feature maps with a size of 1×*H*×*W*. Second, two feature maps with a size of *C*×1×1 were merged by using element-wise summation after entering a weight-sharing network composed of a multi-layer perceptron (MLP) with one hidden layer, and then the sigmoid activation operation was performed to generate the final channel attention *M_C_* ∈*R^C^* ^×1×1^. Two feature maps with a size of 1×*H*×*W* were convolved after the channel dimension splicing, and then the sigmoid activation operation was performed to generate the final spatial attention *M_S_* ∈*R* ^1×^ *^H^* ^×^ *^W^*. Then, *M*_C_ and *F* were merged by using element-wise multiplication to obtain the feature map *F* ' after channel attention adjustment, and *M_S_* and *F* were merged by using element-wise multiplication to obtain the feature map *F* '' after spatial attention adjustment. Finally, we added the feature maps *F* ' and *F* '' to get the input *F* ''' of the *C*4 layer. The calculation process of parallel connected CBAM generating attention is shown in formula (2).


(2) $$F'''=M_{C}(F)⊗F+M_{S}(F)⊗F$$

Where *F* is the input feature map, *F* ''' is the output feature map, *M_C_* (*F*) is the output of CAM, *M_S_* (*F*) is the output of SAM, and ⨂ is the element-wise multiplication (multiply the elements at the corresponding positions of two matrices).

(3) FPN

In the improved Mask RCNN backbone network, the image passed through the bottom-up ResNeXt fused with the attention mechanism to obtain 4-level feature maps (*D*2, *D*3, *D*4, and *D*5) from low to high. Then, these feature maps were used as the input of FPN to establish a feature pyramid network and output new features (*P*2, *P*3, *P*4, *P*5, and *P*6). *P*6 was obtained by the maximum pooling operation of P5. The specific corresponding relationship between feature maps is shown in formula (3).


(3) $$\left\{ \begin{array}{l} P2\;=\;conv\left(sum\left(upsample\left(\text{P}3\right),\;conv\left(D2\right)\right)\right)\\ P3\;=\;conv\left(sum\left(upsample\left(\text{P}4\right),\;conv\left(D3\right)\right)\right)\\ P4\;=\;conv\left(sum\left(upsample\left(\text{P}5\right),\;conv\left(D4\right)\right)\right)\\ P5\;=\;conv(conv(D5))\\ P6\;=\mathrm{maxpooling}\text{(P5)} \end{array}\right.$$

Where *conv* is the convolution operation, *sum* is the element-by-element alignment operation, *upsample* is the up sampling operation, and maxpooling is the max pooling operation.

FPN adopted the convolution layer with a convolution kernel of 1×1 and the top-down and horizontal connection methods to fuse the 4-level feature maps generated by the ResNeXt fused with the attention mechanism. After fusion, each feature map (*P*2, *P*3, *P*4, *P*5, and *P*6) had different levels of features.

#### 2.3.2 RPN and RoIAlign

RPN generated sliding windows of various sizes on the feature map obtained by the backbone network. The sliding windows slid through convolution and selected multiple candidate targets on the feature map. Then, the classifier and regression determined whether the target belonged in the foreground or background and determined the best candidate box position. After obtaining the candidate box, the RoIAlign layer pooled the corresponding area in the feature map into a fixed-size feature map according to the position coordinates of the candidate box to input the fully connected network for classification, bounding box regression, and mask prediction.

The anchor box ratio of the original Mask RCNN network is 0.5:1:2. However, the aspect ratio of these anchor boxes does not match the shape of the tapped area of natural rubber trees, which will reduce the detection and segmentation accuracy of the tapped area of natural rubber trees. Therefore, the aspect ratio of the anchor box needs to be adaptively modified to match the shape of the anchor box with the shape of the tapped area of natural rubber trees. To obtain statistics on the aspect ratio of the tapped area of natural rubber trees, 2000 natural rubber tree images were randomly selected from the sample images, and the tapped area on the 2000 images was manually marked with a rectangular box, and then the aspect ratio of the marked box was counted. The statistical results are shown in **Figure 3C**. In the figure, the abscissa represents the aspect ratio, and the ordinate represents the number of the tapped area corresponding to the corresponding aspect ratio. It can be seen from the figure that the aspect ratio of the tapped area of natural rubber trees was more than 90% between 1 and 2. To adapt to the aspect ratio of the tapped area of most natural rubber trees, the anchor box ratio was set to 1:1.5:2 in this study.

#### 2.3.3 Three-branch network and loss function

The three-branch network is used to obtain the bounding box, category, and mask of the tapped area. In the original Mask RCNN network, the three-branch network inputs the feature map output by the RoIAlign to the FC layers for the classification and bounding box regression of the tapped area and inputs it to the FCN layer for the segmentation of the tapped area. Classification and bounding box regression are implemented by 7×7 convolution operations and two fully connected layers with 1024 feature vectors, and the mask is implemented by four consecutive convolutional layers and one de-convolutional layer. Among them, the kernel size of each convolutional layer is 3×3 with 256 channels.

**Figure 4:**
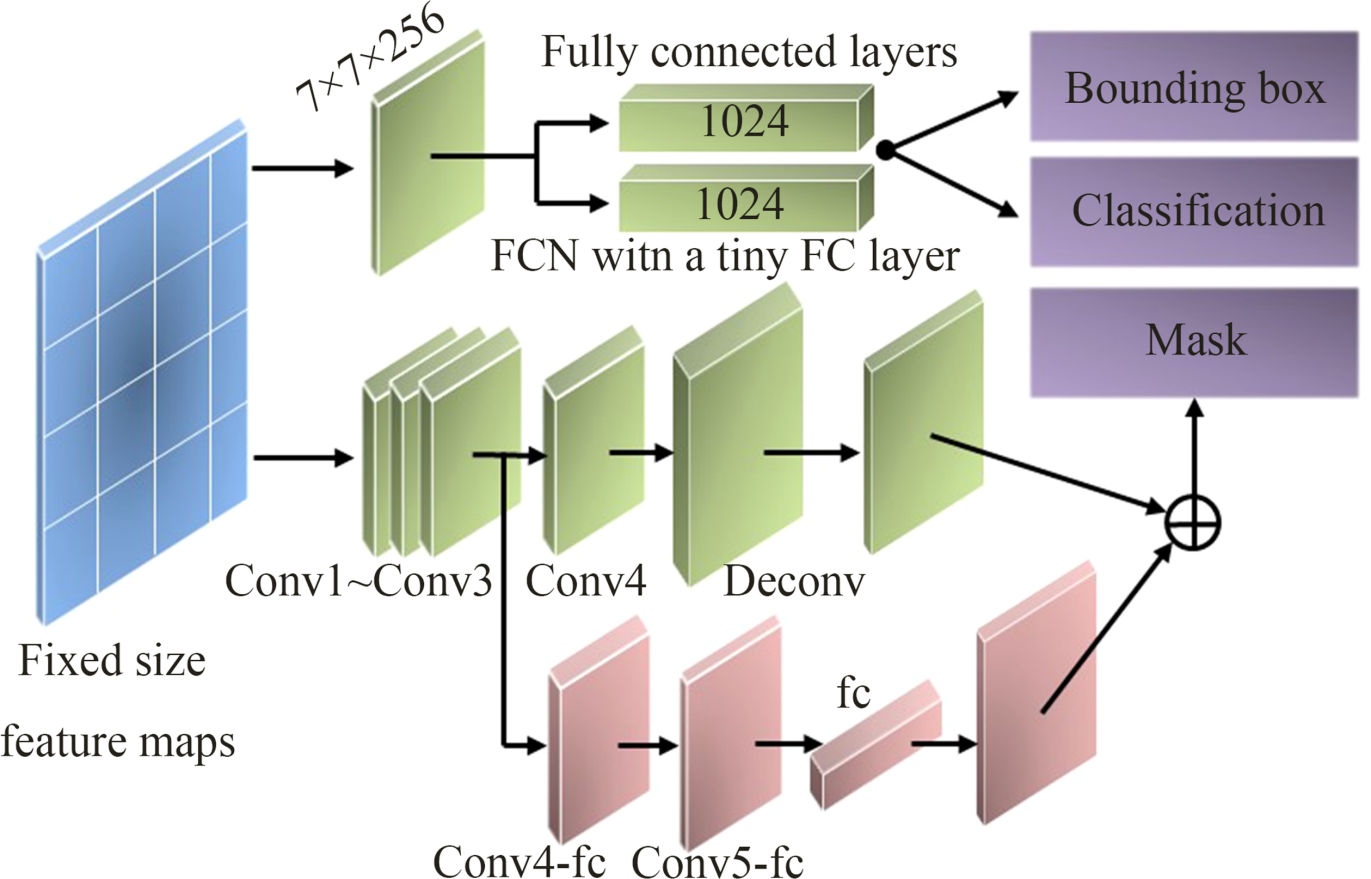
Improved three-branch network.

In this paper, to increase the diversity of information, achieve feature enhancement, and generate a better quality mask effect, a tiny FC layer branch was added to the mask branch, which was connected from conv3 to the fc layer by a branch, passing two conv4_fc and conv5_fc with a 3×3 size of 256 channels, as shown in **Figure 4**. Among them, the number of channels in the conv5_fc convolutional layer was halved to reduce the amount of computation. The 784×1×1 vector generated by the fc layer was reshaped to the same spatial size as the mask predicted by FCN, and the output of a tiny FC layer was added to the output of FCN to obtain the final mask prediction. The fully connected layer and the original FCN had complementary characteristics, which were used to predict unknown background or foreground, had high efficiency and strong generalization ability, and avoided the hiding of spatial features by using a fully connected layer (Wang et al., 2021).

The loss function of the three-branch network is shown in formula (4).


(4) $$L=L_{cls}+L_{box}+L_{mask}$$

Where *L* is the total loss function of the network, *L_mask_* is the mask loss function, *L_cls_* is the classification loss function, and *L_box_* is the bounding box regression loss function. In view of the actual situation of this study, the number of categories is set at 2 (tapped area class and background class).

### 2.4 Fine segmentation of existing tapping line based on edge detection and logic operation

**Figure 5:**
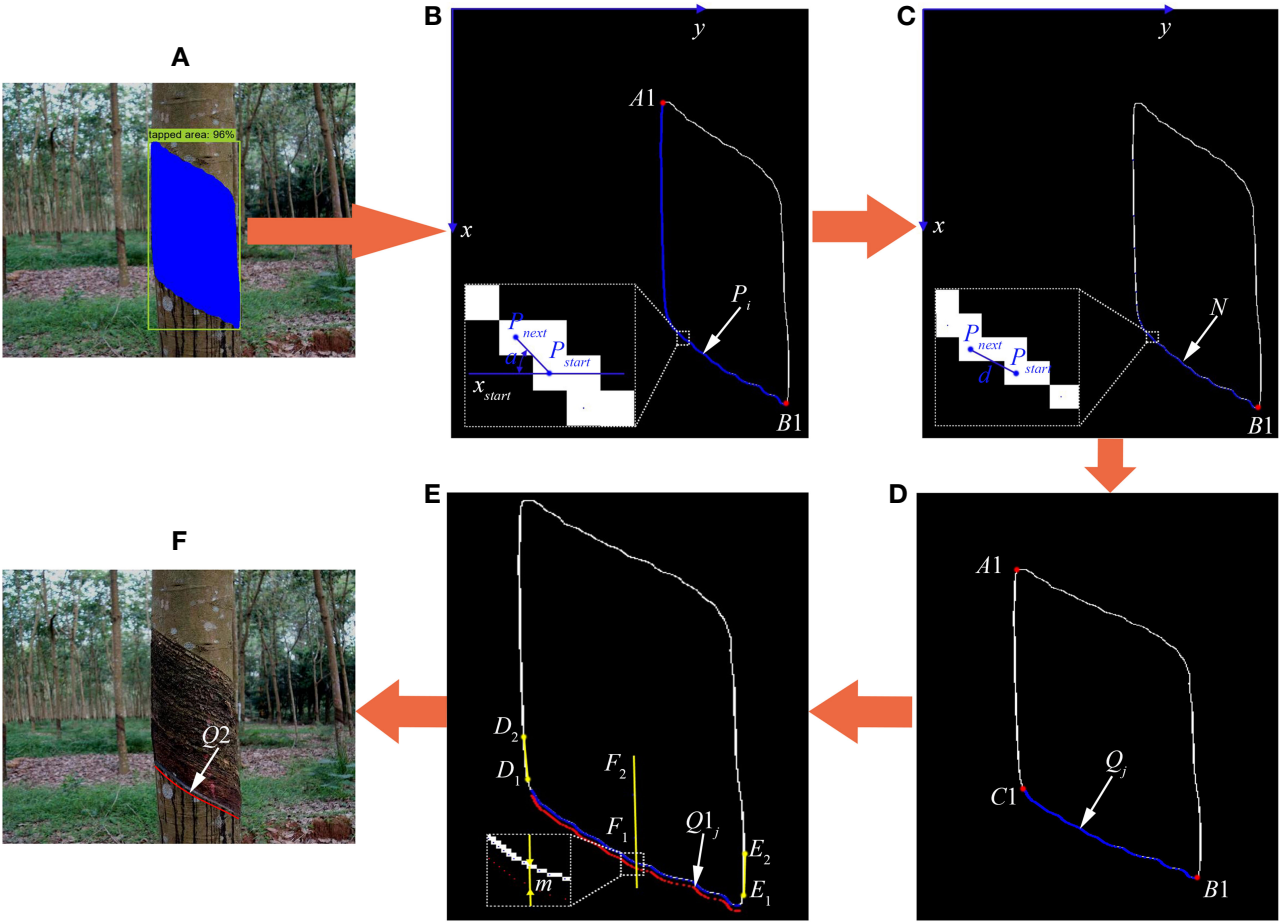
Fine segmentation of existing tapping line and location process of new tapping line. **(A)** Prediction image. **(B, C)** Edge line images of tapped area of natural rubber tree. **(D)** Existing tapping line image of natural rubber tree. **(E)** Schematic diagram of position calculation of new tapping line. **(F)** New tapping line image of natural rubber tree.

The fine segmentation of the existing tapping line is an important prerequisite for the location of the new tapping line. To accurately obtain the existing tapping line, this research used the Canny algorithm based on edge detection (Wang et al., 2022) and logic operation to finely segment the existing tapping line on the basis of improved Mask RCNN’s tapped area detection and rough segmentation. The segmentation process is shown in **Figures 5A–D**, and the specific implementation steps are as follows:

Step 1: Input the image into the improved Mask RCNN model to generate a prediction map (**Figure 5A**) and obtain the rough segmentation results and position information of the natural rubber tree’s tapped area.

Step 2: The obtained rough segmentation result image of the tapped area of the natural rubber tree was converted into a gray-scale image, and the gray-scale image was smoothed by Gaussian filtering.

Step 3: The gradient amplitude and angle of a pixel point of the gray-scale image through the gradient in the *x* and *y* directions were calculated, as shown in formulas (5)-(8). The smoothed gray-scale image was subjected to non-maximum signal suppression processing based on the calculated gradient amplitude and angle.


(5) $$G_{x}(x,y)=\frac{P(x,y+1)-P(x,y)+P(x+1,y+1)-P(x+1,y)}{2}$$

(6) $$G_{y}(x,y)=\frac{P(x,y)-P(x+1,y)+P(x,y+1)-P(x+1,y+1)}{2}$$

(7) $$S=\sqrt{G_{x}^{2}(x,y)+G_{y}^{2}(x,y)}$$

(8) $$\theta =\arctan(\frac{G_{y}(x,y)}{G_{x}(x,y)})$$

Where *G_x_* (*x*, *y*) is the gradient of the image in the *x* direction, *G_y_* (*x*, *y*) is the gradient of the image in the *y* direction, *P*(*x*, *y*) is the pixel value of the image at a certain point, *S* is the gradient magnitude of the pixel point, and *θ* is the angle of the pixel point.

Step 4: After double threshold edge connection processing was performed on the image obtained in step 3, binarization was performed, and the result was output to obtain the edge line and position information of the tapped area of the natural rubber tree.

Step 5: The upper left extreme point *A*1 (*x_A_*, *y_A_*) (**Figure 5B**) and the lower right extreme point *B*1 (*x_B_*, *y_B_*) (**Figure 5B**) of the edge line of the tapped area were calculated. Starting from point *B*1, along the ordinate direction from point *B*1 to point *A*1, that is, from *x_B_* to *x_A_*, find the minimum value *y_i_* of the abscissa corresponding to each ordinate *x_i_* until it reaches point *A*1. And then the minimum value *y_i_* of the abscissa corresponded to each ordinate *x_i_* was corresponded to *x_i_* one by one to obtain the coordinate set of each point on the lower left edge line of the tapped area. Among them, *i* = 1, 2,…, *n*; *B*1 = *P*_1_, and *B*1 is the end point of the existing tapping line.

Step 6: The first 50 points of the point set *P_i_* were saved in the point sets *N* and *Q*, and the 50th point of the point set *P_i_* was set as the initial point *P_start_*.

Step 7: Starting from the initial point *P_start_*, we judged whether the angle *a* (**Figure 5B**) between the line segment formed by the initial point *P_start_* and the next point *P_next_* and the abscissa *x_star_* satisfied the angle constraint 0 ≤ *a* ≤ *π*/2 in the point set *P_i_*. If so, the next point *P_next_* was saved in the point set *N* and step 7 continued to be performed until the point *P_n_* was reached.

Step 8: Starting from the initial point *P_start_*, we judged whether the distance *d* (**Figure 5C**) between the initial point *P_start_* and the next point *P_next_* satisfied the distance constraint *d* ≤ 1.5 in the point set *N*. If so, the next point *P_next_* was saved in the point set *Q*, and step 8 was continued to be performed until the distance between the two points *P_start_* and *P_next_* did not meet the distance constraint and the existing tapping line points *Q_j_* (*x_j_*, *y_j_*) (**Figure 5D**) of the natural rubber tree were obtained. Among them, *j* = 1, 2,…, *n*1; *C*1 = *Q_n_*_1_, and *C*1 is the starting point of the existing tapping line.

### 2.5 Position calculation of new tapping line

During the rubber tapping operation of natural rubber trees, to ensure rubber production, reasonably plan the tapping area, and reduce the dead skin rate of rubber trees, the new tapping line is generated by moving the existing tapping line down a certain distance along the center line of the left and right edge lines of the tapped area. The implementation process is shown in **Figures 5D–F**, and the specific solution process is as follows:

#### 2.5.1 Obtain center line of left and right edge lines of tapped area

The center line of the left and right edge lines of the tapped area is obtained based on the starting point and end point of the existing tapping line and the edge line of the tapped area. The specific calculation steps are as follows:

First, starting from point *C*1 (**Figure 5D**), along the ordinate direction from point *C*1 to point *A*1 (**Figure 5D**), that is, from *x_C_* to *x_A_*, the minimum values of the abscissa corresponding to the ordinates *x_C_*–30 and *x_C_*–60 were obtained to form coordinate points *D*_1_ (*x_D_*_1_, *y_D_*_1_) and *D*_2_ (*x_D_*_2_, *y_D_*_2_), and the line segment *D*_1_ *D*_2_ (**Figure 5E**) was taken as the left edge line of the tapped area. Then, starting from point *B*1 (**Figure 5D**), along the ordinate direction from point *B*1 to point *A*1 (**Figure 5D**), that is, from *x_B_* to *x_A_*, the maximum values of the abscissa corresponding to the ordinates *x_B_*–30 and *x_B_*–60 were obtained to form coordinate points *E*_1_ (*x_E_*_1_, *y_E_*_1_) and *E*_2_ (*x_E_*_2_, *y_E_*_2_), and the line segment *E*_1_ *E*_2_ (**Figure 5E**) was taken as the right edge line of the tapped area. Finally, the center line *F*_1_ *F*_2_ (**Figure 5E**) of the left and right edge lines of the tapped area was calculated by the formula (9).


(9) $$M=\left\{ \begin{array}{l} \left(x_{F1},y_{F1}\right)=\left(x_{D1},y_{D1}\right)+\frac{\left(x_{E1},y_{E1}\right)-\left(x_{D1},y_{D1}\right)}{2}\\ \left(x_{F2},y_{F2}\right)=\left(x_{D2},y_{D2}\right)+\frac{\left(x_{E2},y_{E2}\right)-\left(x_{D2},y_{D2}\right)}{2} \end{array}\right.$$

Where *M* denotes the center line *F*_1_ *F*_2_, (*x_F_*_1_, *y_F_*_1_) denotes the coordinates of the lower endpoint of the center line *F*_1_ *F*_2_, and (*x_F_*_2_, *y_F_*_2_) denotes the coordinates of the upper endpoint of the center line *F*_1_ *F*_2_.

#### 2.5.2 Determination position of new tapping line

It was known that the coordinates of each point of the existing tapping line were *Q_j_* (*x_j_*, *y_j_*), and the coordinates *Q*1*_j_* (*x*1*_j_*, *y*1*_j_*) (**Figure 5E**) of each point of the new tapping line could be calculated by the formula (10). Among them, *j* = 1, 2,…, *n*1.


(10) $$\left\{ \begin{array}{l} \frac{y1_{j}\;-\;y_{j}}{x1_{j}\;-\;x_{j}}=\frac{y_{F1}\;-\;y_{F2}}{x_{F1}\;-\;x_{F2}}\\ \sqrt{{\left(x1_{j}-x_{j}\right)}^{2}+{\left(y1_{j}-y_{j}\right)}^{2}}=m \end{array}\right.$$

Where *m* is the number of pixels that the existing tapping line moves down along the center line of the left and right edge lines of the tapped area and is also the bark consumption along the trunk axis direction of the natural rubber tree during the rubber tapping operation. *m* is 8 here.

After obtaining the coordinates *Q*1*_j_* (*x*1*_j_*, *y*1*_j_*) of each point of the new tapping line, the cubic polynomial was used to fit the curve of the new tapping line (Zhang et al., 2020), eliminate redundant points, realize smooth processing of the new tapping line, and form a new tapping path that was conducive to the execution of the end effector of the rubber tapping robot.

The mathematical expression of the polynomial curve is given in formula (11).


(11) $$p_{n}(y)=\sum_{k=0}^{n}a_{k}y^{k}$$

Where *n* is the degree and *a_k_* is the polynomial coefficient. *a_k_* is obtained by substituting the coordinates *Q*1*_j_* of each point of the new tapping line into formula (12) and then substituting the obtained *a_k_* into formula (11) to generate a smooth new tapping line *Q*2 (**Figure 5F**).


(12) $$\left[\begin{array}{cccc} m+1 & \sum_{j=0}^{m}y_{j} & \cdots & \sum_{j=0}^{m}y_{j}^{n}\\ \sum_{j=0}^{m}y_{i} & \sum_{j=0}^{m}y_{j}^{2} & \cdots & \sum_{j=0}^{m}y_{j}^{n+1}\\ \vdots & \vdots & \vdots \\ \sum_{j=0}^{m}y_{j}^{n} & \sum_{j=0}^{m}y_{j}^{n+1} & \cdots & \sum_{j=0}^{m}y_{j}^{2n} \end{array}\right]\left[\begin{array}{c} a_{0}\\ a_{1}\\ \vdots \\ a_{n} \end{array}\right]=\left[\begin{array}{c} \sum_{j=0}^{m}x_{j}\\ \sum_{j=0}^{m}y_{j}x_{j}\\ \vdots \\ \sum_{j=0}^{m}y_{j}^{n}x_{j} \end{array}\right]$$

Where *m* is the total number of points on the new tapping line, *x* is the abscissa of each point on the new tapping line, and *y* is the ordinate of each point on the new tapping line.

### 2.6 Model training and algorithm performance evaluation

The network model training and the new tapping line location algorithm test of this study were carried out on two computers, respectively. The model training was carried out on the hardware platforms of an Intel (R) Xeon (R) Silver 4210 processor, 32GB memory, and an NVIDIA RTX 4000 (8GB memory). The detection and segmentation of the tapped area of natural rubber trees and the location test of the new tapping line were carried out on the hardware platform of an Intel Core i7-11800H processor, 16GB memory, and an NVIDIA RTX 3050 (4GB memory). The software environment used in the experiment was a Windows 64-bit system, the PyTorch deep learning framework, and the Python programming language.

#### 2.6.1 Network model training

To verify the detection and segmentation effects of the improved network model and determine the optimal network model, six models of two different backbone networks were used for comparative experiments, namely the original Mask RCNN model based on ResNet50 and ResNet101, the improved Mask RCNN model based on ResNeXt50 and ResNeXt101 fused with the attention mechanism, and the improved Mask RCNN model based on ResNeXt50 and ResNeXt101 fused with the attention mechanism, which changed the structural parameters and mask branches. Based on the above comparative experiments, the optimal Mask RCNN network model was determined, and the comparative experiments were carried out with YOLACT, Cascade Mask RCNN, PointRend, Swin-B Cascade Mask RCNN, FCN, and DeepLabv3 to compare the detection and segmentation performance of different models and further verify the detection and segmentation effect of the improved model. All of the above model training’s learning rate, batch size, momentum factor, weight decay, and number of iterations were set to 0.01, 2, 0.9, 0.0001, and 30 epochs, respectively.

#### 2.6.2 Model evaluation metrics

To test the performance of the algorithm proposed in this study, 560 images of the test set were tested and evaluated for the detection of the tapped area and the location of the new tapping line. The precision (*P*, %), recall (*R*, %), F1 score (F1, %), average precision (AP, %), and intersection over union (IOU, %) were used as indicators to evaluate the effectiveness of the model, and the location success rate (*Y*, %) of the new tapping line was counted. *P*, *R*, F1, AP, IOU, and *Y* are calculated as follows:


(13) $$p=\frac{TP}{TP+FP}\times 100\%$$

(14) $$R=\frac{TP}{TP+FN}\times 100\%$$

(15) $$\text{F}1=\frac{2\times p\times R}{p+R}$$

(16) $$\text{AP}=\int_{0}^{1}p(R)dR$$

(17) $$\text{IOU}=\frac{M_{TP}}{M_{TP}+M_{FP}+M_{FN}}$$

(18) $$Y=\frac{S_{T}}{S}\times 100\%$$

Where *T_p_*, *F_p_*, and *F_N_* represent true positive, false positive, and false negative, respectively. F1 is the harmonic mean value of the *R* and *P*; the value range is 0 to 1, where 1 represents the best model output and 0 represents the worst model output. AP is the integral of the *P* on the *R*. Generally, the higher the AP value, the better the model’s performance. *M_TP_*, *M_FP_*, and *M_FN_* represent the number of correct divided pixels, wrong divided pixels, and miss divided pixels, respectively. *S* is the number of the existing tapping line, and *S_T_* is the number of the new tapping line’s location success.

## 3 Results and discussion

### 3.1 Improved Mask RCNN for natural rubber tree tapped area detection and segmentation

**Figure 6:**
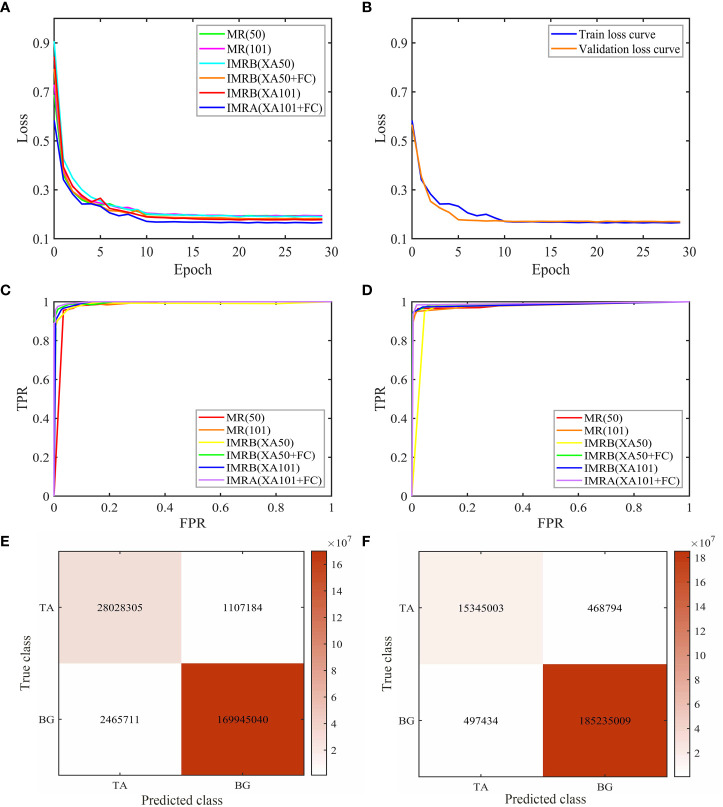
Comparative experiment results of six models. **(A)** Loss curves of different models in the training stage. **(B)** Loss curves of IMRA(XA101+FC). **(C)** Detection ROC curves of different models. **(D)** Segmentation ROC curves of different models. **(E)** Detection confusion matrix of IMRA(XA101+FC). **(F)** Segmentation confusion matrix of IMRA(XA101+FC). (“tapped area”: TA, “background”: BG).

**Figure 7:**
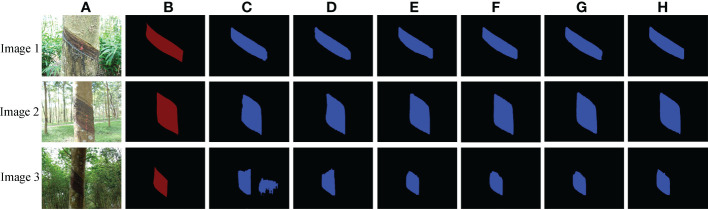
Tapped area segmentation results of natural rubber trees of different models. **(A)** Original images. **(B)** Label images. **(C)** MR(50). **(D)** MR(101). **(E)** IMRB(XA50). **(F)** IMRB(XA101). **(G)** IMRA(XA50+FC). **(H)** IMRA(XA101+FC).

In order to verify the detection and segmentation performance of the improved Mask RCNN model proposed in this paper, six models with two different backbone networks were compared. The comparative experiments were divided into training and testing stages. The loss functions of the six models are compared in the training stage, as shown in **Figures 6A, B**. In the test stage, 560 natural rubber tree images from the test set were used to test six models. The results are shown in **Table 1**, **Figures 6C–F**, and **Figure 7**.

**Table 1:** Performance results of different models in the test set.

Model	AP_0.5_	AP_0.75_	AP_0.5-0.95_	IOU	Parameters (M)	Time (s)
MR (50)	97.2	89.6	75.02	87.9	43.92	0.097
MR (101)	97.58	91.48	75.78	89.09	62.86	0.130
IMRB (XA50)	99.18	93	75.59	91.15	43.55	0.129
IMRB (XA101)	99.47	97.39	77.28	92.19	62.63	0.174
IMRA (XA50+FC)	99.16	95.98	75.77	91.72	44.73	0.129
IMRA (XA101+FC)	99.6	97.62	80.59	93.71	63.81	0.173

MR is the Mask RCNN network model. IMRB is the improved Mask RCNN network model that changes the backbone network. IMRA is the improved Mask RCNN network model that changes the backbone network, structural parameters, and mask branches. 50 is the ResNet50. 101 is the ResNet101. XA50 is the ResNeXt50 fused with attention mechanism. XA101 is the ResNeXt101 fused with attention mechanism. FC is a tiny fully connected layer branch. AP_0.5_ is the average precision when the IOU threshold is greater than 0.5, AP_0.75_ is the average precision when the IOU threshold is greater than 0.75, and AP_0.5-0.95_ is the average precision when the IOU threshold is between 0.5 and 0.95. Time is the total time spent on detecting and segmenting a single image.

According to **Figures 6A, B**, during the training stage, the total loss value of the six models gradually decreased and tended to be stable with the increase in the number of epochs, and the decline speed was the fastest within the range of 0 to 10 epochs. The network reached a convergence state when the number of epochs was increased from 15 to 29. The total loss value of the training tended to be stable and did not change much. Among them, the overall training loss value of the IMRA(XA101+FC) model was lower than the other models, indicating that the IMRA(XA101+FC) model utilized image features more thoroughly than the other five models and showed better performance (Tian et al., 2020). In addition, with the increase in the number of epochs, the training set loss value and the test set loss (validation loss) value of the IMRA(XA101+FC) model decreased continuously. When the number of epochs was greater than 15, the training set loss value and the test set loss value gradually converged. The loss values were less than 0.2 and tended to be stable around 0.17. This indicated that the IMRA(XA101+FC) model had a good training effect.

In the testing stage, it can be seen from **Table 1** that compared with MR(50), the AP_0.5_, AP_0.75_, and AP_0.5-0.95_ of IMRB(XA50) and IMRA(XA50+FC) increased by 1.98%, 3.4%, 0.57%, and 1.96%, 6.35%, 0.75%, respectively. Compared with MR(101), the AP_0.5_, AP_0.75_, and AP_0.5-0.95_ of IMRB(XA101) and IMRA(XA101+FC) increased by 1.89%, 5.91%, 1.5%, and 2.02%, 6.14%, 4.81%, respectively. The AP values of MR(101), IMRB(XA101), and IMRA(XA101+FC) were higher than MR(50), IMRB(XA50), and IMRA(XA50+FC), indicating that compared with ResNet50, ResNet101, and ResNeXt50 fused with the attention mechanism, the ResNeXt101 fused with the attention mechanism had a strong ability to extract features, and the object detection performance of the model was significantly improved (Panta et al., 2020; Du et al., 2022; Ma et al., 2022). The AP_0.5-0.95_ of IMRA(XA50+FC) and IMRA(XA101+FC) with changing structural parameters and mask branches increased by 0.18% and 3.31%, respectively, when compared to IMRB(XA50) and IMRB(XA101). It showed that IMRA(XA101+FC), which modified the anchor box parameters and added a tiny FC layer to the mask branch, paid more attention to the object itself and had the best object detection performance (Wang et al., 2021).

In terms of image segmentation performance, the network model of Mask RCNN using ResNeXt fused with the attention mechanism as the backbone network was significantly superior to the Mask RCNN model using ResNet as the backbone network in the IOU index, and the IOU values of IMRA(XA50+FC) and IMRA(XA101+FC) were increased by 0.57% and 1.52%, respectively, compared with IMRB(XA50) and IMRB(XA101). It showed that a tiny FC layer branch was added to the mask branch to further increase the information diversity, realize feature enhancement, and improve the segmentation performance of the model (Wang et al., 2021). When combined with **Figure 7**, it is clear that IMRA(XA101+FC) had a better segmentation effect than other models, and its segmentation results were more accurate. Among them, the segmentation of the tapped area of the deeper backbone network fused with the attention mechanism was more accurate, and after a tiny FC layer branch was added to the mask branch, the diversity of information was increased and a better quality mask was generated, as shown in **Figure 7H**.

In addition, ROC curves and confusion matrices were used to summarize the detection and segmentation performance of IMRA (XA101+FC). It can be seen from **Figures 6C, D** that, in the test stage, the detection and segmentation ROC curve of IMRA(XA101+FC) was the most convex, closest to the upper left corner, and the area under the curve was the largest compared with ROC curves of other models. It showed that, compared with other models, IMRA(XA101+FC) had the highest detection and segmentation accuracy and the best detection and segmentation performance. As shown in **Figures 6E, F**, diagonal lines in the matrix were correctly detected and segmented, while all other items were incorrectly detected and segmented. From **Figures 6E, F**, it can be seen that the accuracy of detection and segmentation of the tapped area of natural rubber trees reached 98.23% and 99.52%, respectively.

In the total time spent on detection and segmentation, IMRA(XA50+FC) and IMRB(XA50) were 0.032 s slower than MR(50), while IMRA(XA101+FC) and IMRB(XA101) were 0.044 s and 0.043 s slower than MR(101), which indicated that the ResNeXt50 and ResNeXt101 fused with the attention mechanism had slowed down the detection and segmentation speed of the Mask RCNN model to a certain extent, but the impact was not very obvious. IMRB(XA50) spent the same time as IMRA(XA50+FC), while IMRA(XA101+FC) was 0.001 s faster than IMRB(XA101), which indicated that changing the anchor box ratio and adding a tiny FC layer branch into the mask branch had a weak impact on the model detection and segmentation speed. In terms of model parameters, IMRB(XA50) and IMRB(XA101) decreased by 0.37 and 0.23, respectively, compared with MR(50) and MR(101). When compared to MR(50) and MR(101), IMRA(XA50+FC) and IMRA(XA101+FC) increased by 0.81 and 0.95, respectively. It showed that ResNeXt50 and ResNeXt101, which fused with the attention mechanism, reduced the parameters of the Mask RCNN model. However, modifying the anchor box parameters and adding a tiny FC layer to the mask branch increased the parameters of the Mask RCNN to a certain extent, but the impact was not significant.

To sum up, all the detection and image segmentation indexes of IMRA(XA101+FC) were optimal in both the training and testing stages. The detection and segmentation accuracy of IMRA(XA101+FC) reached 98.23% and 99.52%, respectively. Compared with MR(101), AP_0.5_, AP_0.75_, AP_0.5-0.95_, and IOU were increased by 2.02%, 6.14%, 4.81%, and 4.62%, respectively. Although IMRA(XA101+FC) was 0.043 s slower than MR(101) in detection and segmentation speed, and 0.95 M more than MR (101) in model parameters, it could meet the task requirements. In addition, considering that the fine segmentation of the existing tapping line and the location of the new tapping line were based on the detection and segmentation results of the tapped area. Therefore, IMRA(XA101+FC) was determined as the improved Mask RCNN network model, MR(101) was the Mask RCNN network model, and the improved Mask RCNN network model was used as the pre-network for the fine segmentation of the existing tapping line.

### 3.2 Detection and segmentation performance of natural rubber tree tapped area based on different methods

**Figure 8:**
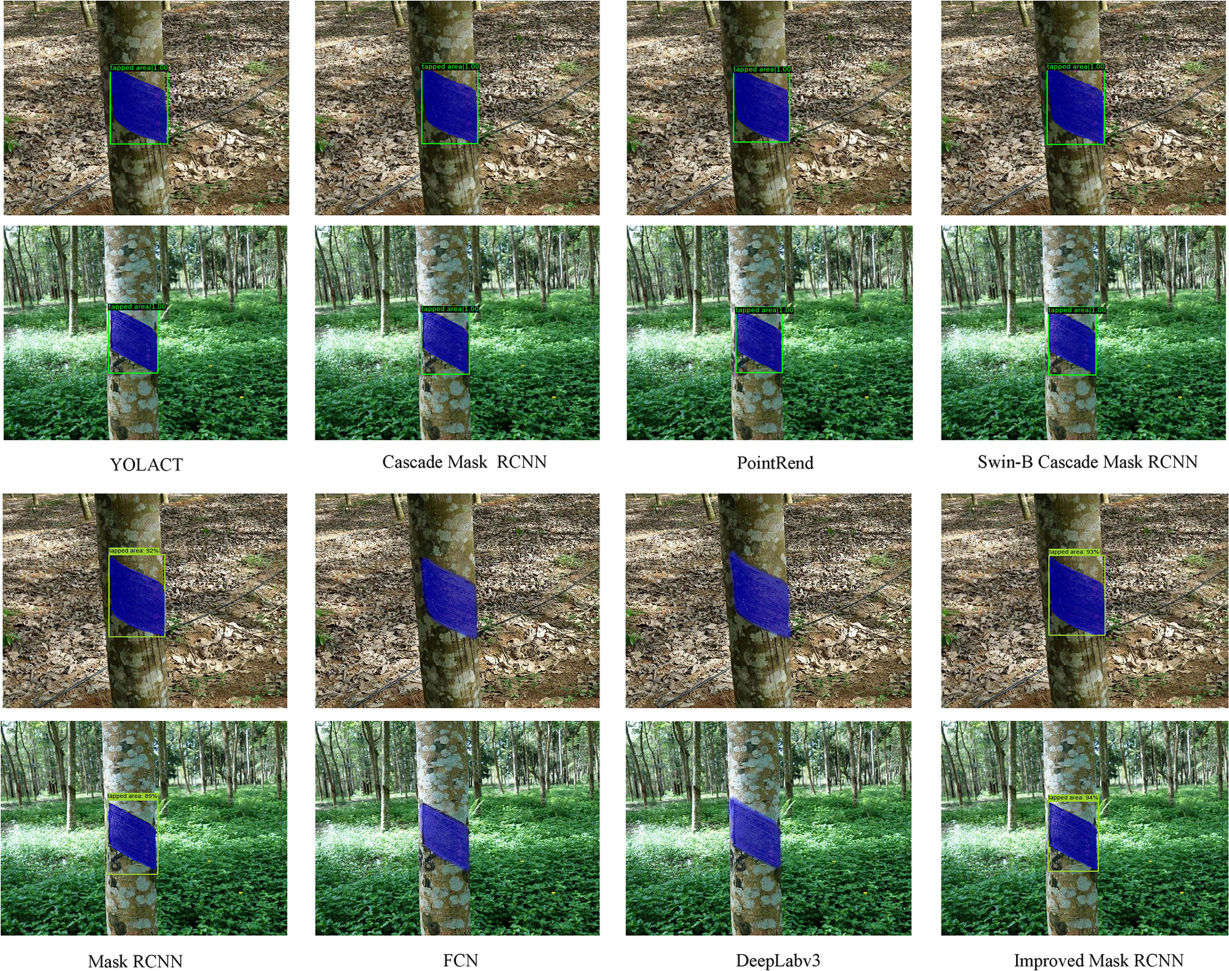
Tapped area detection and segmentation results of natural rubber trees of different models.

To further verify the detection and segmentation performance of the improved Mask RCNN network model on the tapped area of natural rubber trees, it was compared with the Mask RCNN, YOLACT, Cascade Mask RCNN, PointRend, Swin-B Cascade Mask RCNN, FCN, and DeepLabv3 models (He et al., 2017; Shelhamer et al., 2017; Bolya et al., 2019; Cai and Vasconcelos, 2019; Yurtkulu et al., 2019; Kirillov et al., 2020; Liu et al., 2021). The test results of the improved Mask RCNN network model and the other models on the tapped area detection and segmentation of 560 natural rubber tree images in the test set are shown in **Table 2** and **Figure 8**.

**Table 2:** Detection and segmentation performance of different models on tapped area in test set.

Model	YOLACT	Cascade Mask RCNN	PointRend	Swin-B Cascade Mask RCNN	Mask RCNN	FCN	DeepLabv3	Improved Mask RCNN
AP_box_	98.8	98.8	99	98.9	97.58	/	/	99.6
AP_mask_	98.8	98.8	99	98.9	96.97	99.11	97.31	99.78

AP_box_ is the average precision of natural rubber tree tapped area detection. AP_mask_ is the average precision of natural rubber tree tapped area segmentation. / indicates that the average precision value of natural rubber tree tapped area detection does not exist.

It can be seen from **Table 2** that the AP_box_ and AP_mask_ values of the improved Mask RCNN reached 99.6% and 99.78%, respectively. Compared with YOLACT, Cascade Mask RCNN, PointRend, Swin-B Cascade Mask RCNN, and Mask RCNN, the AP_box_ value of the improved Mask RCNN increased by 0.8%, 0.8%, 0.6%, 0.7%, and 2.02%, respectively, and the AP_mask_ value of the improved Mask RCNN increased by 0.98%, 0.98%, 0.78%, 0.88%, and 2.81%, respectively. Compared with FCN and DeepLabv3, the AP_mask_ value of the improved Mask RCNN increased by 0.67% and 2.47%, respectively. This indicated that, among these models, the improved Mask RCNN model had the best detection and segmentation performance.

As can be seen from **Figure 8**, compared with the improved Mask RCNN, the detection and segmentation effects of YOLACT, Cascade Mask RCNN, PointRend, Swin-B Cascade Mask RCNN, FCN, DeepLabv3, and Mask RCNN models were not ideal. For example, FCN and DeepLabv3 had the phenomenon of object over-segmentation; Mask RCNN had the phenomenon of object incomplete segmentation and object over-detection; Cascade Mask RCNN and Swin-B Cascade Mask RCNN had the phenomenon that the object edge segmentation was not smooth; PointRend had the phenomenon of object incomplete detection; and YOLACT had the phenomenon of object incomplete detection and segmentation. However, the improved Mask RCNN still maintained a good detection and segmentation effect.

Through comprehensive comparison, the improved Mask RCNN model had higher values in AP_box_ and AP_mask_ and had a better detection and segmentation effect on the tapped area of natural rubber trees, which indicated that the improved Mask RCNN had better detection and segmentation performance and that the network’s accuracy, robustness, and generalization performance were better.

### 3.3 Comparison of natural rubber tree tapped area detection effects under different shooting conditions

**Figure 9:**
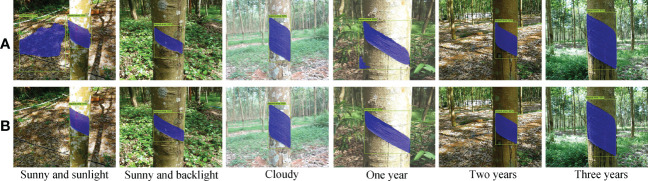
Tapped area detection and segmentation results of natural rubber trees of different models under different shooting conditions. **(A)** Mask RCNN. **(B)** Improved Mask RCNN.

To further verify the detection effect of the improved Mask RCNN network model on the tapped area of natural rubber trees under different shooting conditions, the 560 images of the test set were divided into direct sunlight on sunny days, backlight on sunny days, cloudy days, the tapped area had been cut for one year, the tapped area had been cut for two years, and the tapped area had been cut for three years. The comparative experiment of the detection effect before and after the model improvement was carried out on the divided test set (Ning et al., 2021). The specific effects are shown in **Figure 9** and **Table 3**.

**Table 3:** Detection results before and after model improvement under different shooting conditions.

Shooting condition	Before and after the model improvement	*P*	*R*	F1	IOU
Sunny and sunlight	Before	89.69	98.06	92.43	88.01
	After	95.09	97.98	96.45	93.25
Sunny and backlight	Before	92.1	97.23	93.93	89.69
	After	94.99	97.19	96.02	92.44
Cloudy	Before	92.62	98.61	95.13	91.42
	After	95.35	98.7	96.98	94.16
A year	Before	89.27	97.33	91.97	87.03
	After	94.3	96.68	95.41	91.33
Two years	Before	89.92	97.87	92.52	88.08
	After	94.97	98.14	96.46	93.28
Three years	Before	93.27	98.29	95.38	91.75
	After	95.68	98.38	96.99	94.18

It can be seen from **Figure 9** that under different shooting conditions, the unimproved model had a false detection phenomenon. It was analyzed that the reason was that the color and shape of the natural rubber tree’s shadow in the rubber garden were too close to the color and shape of the tapped area under direct sunlight on sunny days. However, the improved model could accurately detect the position and category of the tapped area of natural rubber trees without false detection and had higher detection accuracy. In the segmentation of the natural rubber tree tapped area, the unimproved model had the phenomena of incomplete segmentation, over-segmentation, the segmentation boundary was not detailed, and there were burrs. It was analyzed that the appearance of this phenomenon was due to the irregular shape of the natural rubber tree tapped area, uneven tapped area color, uneven illumination, etc. However, the improved model significantly improved this phenomenon, making its segmentation accuracy closer to the real area.

The detection results of the tapped area before and after the model improvement under different shooting conditions are shown in **Table 3**. It can be seen from **Table 3** that under cloudy days, the *P*, *R*, and IOU of the improved model reached 95.35%, 98.7%, and 94.16%, respectively, indicating that the tapped area of natural rubber trees under this condition was easier to detect by the model. However, on sunny days, the *P*, *R*, and IOU of the improved model were lower than those on cloudy days. On cloudy days, the color, texture, and profile of the tapped area of natural rubber trees were clear; improved model detection was less difficult; and detection and segmentation effects were better. However, on sunny days, due to the strong light and uneven light distribution, the tapped area experienced an exposure phenomenon. The shadow generated by the light overlapped with the tapped area’s color, and the color of the tapped area was similar to the background. Therefore, the improved model was more difficult to detect the tapped area under the conditions of direct sunlight and backlight on sunny days, and the detection and segmentation effects were poor. It can be seen from **Table 3** that, under the conditions that the tapped area had been cut for two and three years, the *P*, *R*, and IOU of the improved model were significantly higher than those of the tapped area that had been cut for one year, indicating that the tapped area of natural rubber trees that had been cut for two or three years was easier to detect by the improved model. The reason was that the tapped area of natural rubber trees that had been cut for two or three years was relatively large and conspicuous, which made it easy to detect, and the model detection and segmentation effects were better. However, the tapped area of natural rubber trees that had been cut for one year was smaller and less obvious than the tapped area of natural rubber trees that had been cut for two or three years, which made it difficult to detect, resulting in poor detection and segmentation effects of the improved model. In addition, the F1 and IOU values of the improved model proposed in this study were higher than those of the unimproved model under different shooting conditions, indicating that the detection and segmentation performance of this method was better than that of the unimproved model under different shooting conditions.

Aiming at the situation that the improved model mentioned above has poor detection and segmentation effects under the conditions of direct sunlight on sunny days, backlight on sunny days, and the tapped area has been cut for one year, we will further improve the feature extraction ability of the model by expanding the training dataset in the future to solve this situation.

### 3.4 New tapping line location for natural rubber trees

**Figure 10:**
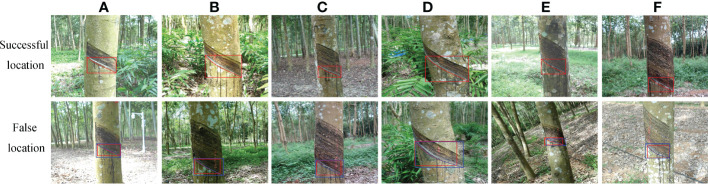
Location effects of new tapping line under different shooting conditions. **(A)** Sunny and sunlight. **(B)** Sunny and backlight. **(C)** Cloudy. **(D)** One year. **(E)** Two years. **(F)** Three years.

To verify the influence of different shooting conditions on the location accuracy and speed of the new tapping line, the images taken under different shooting conditions in the test set were tested. The location effects of the new tapping line are shown in **Figure 10** and **Table 4**.

**Table 4:** Location results of new tapping line under different shooting conditions.

Shooting condition	*S*	*S_T_*	*Y*	Time spent (s)
Sunny and sunlight	296	268	90.54	0.188
Sunny and backlight	188	166	88.93	0.19
Cloudy	76	70	92.11	0.188
A year	130	115	88.46	0.191
Two years	246	222	90.24	0.188
Three years	184	167	90.76	0.19
Mean	560	504	90	0.189

Time spent is the total time of single image object detection, segmentation, and new tapping line location.

It can be seen from **Figure 10** and **Table 4** that under different shooting conditions, the average success rate of the new tapping line location was 90% and the average time spent was 0.189 s, which could meet the task requirements. On cloudy days, the success rate of locating the new tapping line was 92.11%, which was 1.57% and 3.18% higher than that under direct sunlight on sunny days and backlight on sunny days, respectively. On cloudy days, because the color, texture, profile, shape, and other characteristics of the tapped area of natural rubber trees were more obvious, the detection and segmentation accuracy of the improved Mask RCNN network model was higher, resulting in a higher location success rate for the new tapping line. On sunny days, factors such as strong light and uneven light distribution affect the image quality. The most obvious one was the tapped area. As shown in **Figures 10A, B**, the tapped area that failed to be successfully located had an exposure phenomenon, the shape and texture characteristics were fuzzy, and the color was similar to the background. As a result, the improved Mask RCNN failed to effectively detect and segment the tapped area of natural rubber trees, resulting in the new tapping line location failure.

The success rates of the location of the new tapping line of natural rubber trees where the tapped area had been cut for two and three years were 90.24% and 90.76%, respectively, which were higher than those of the tapped area that had been cut for one year. In the images of the natural rubber tree, where the tapped area had been cut for two or three years, the tapped area was a large target, which was more conspicuous and easy to detect, and the image processing process was relatively simple. The tapped area was a small target in the images of the natural rubber tree where the tapped area had been cut for one year, making it difficult to detect, and the segmentation effect was poor. As shown in **Figure 10D**, the segmentation effect of the tapped area was poor, which led to errors in the fine segmentation of the existing tapping line based on edge detection and logic operations, resulting in the failure of the new tapping line to locate.

**Figure 11:**
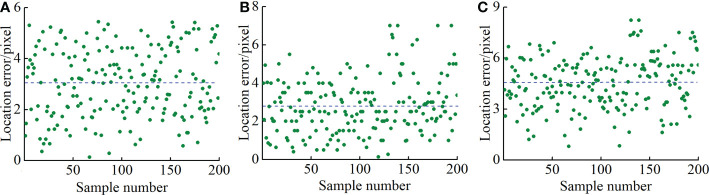
Location error of new tapping line. **(A)** Location error in *x*, **(B)** location error in *y*, and **(C)** total location error.

An error analysis was performed on 200 sample images that successfully located the new tapping line to calculate the location accuracy of the algorithm. Labelme was used to mark the rectangular area [(*X*_min_, *Y*_min_), (*X*_max_, *Y*_max_)] of the optimal new tapping line. The pixel location error was calculated according to the formula (19) (Du et al., 2022). The location error analysis results are shown in **Figure 11**.

It can be seen from **Figure 11** that the maximum values of the location errors in the *x* and *y* directions and the total location error were 5.5, 7, and 8.2 pixels, respectively, and the average errors were 3, 2.8, and 4.5 pixels, respectively. Compared with the average errors, the maximum deviations were 2.5, 4.2, and 3.7 pixels, respectively, which indicated that the location errors in the *x* and *y* directions and the total location error were small and that change was relatively stable compared with the average error. Considering that the rubber tapping operation of natural rubber trees is an operation with high precision requirements, it is required to have a certain fault tolerance range when designing the rubber tapping end effector, and its location tolerance is 10 pixels (Zhang et al., 2019). Therefore, the location results of the new tapping line can meet the location accuracy requirements of natural rubber tree tapping machinery.


(19) $$\left\{ \begin{array}{l} e_{x}=\frac{\left|X_{\min}-x_{\min}\right|+\left|X_{\max}-x_{\max}\right|}{2}\\ e_{y}=\frac{\left|Y_{\min}-y_{\min}\right|+\left|Y_{\max}-y_{\max}\right|}{2}\\ e=\frac{\sqrt{e_{x}^{2}+e_{y}^{2}}}{2} \end{array}\right.$$

Where (*x*_min_, *x*_max_) and (*y*_min_, *y*_max_) are the abscissa and ordinate coordinates of the rectangular area that successfully locates the new tapping line, *e_x_* is the abscissa error, *e_y_* is the ordinate error, and *e* is the total error.

Although this research provided an effective solution for locating the new tapping line of natural rubber trees, it was still affected by many factors, such as the direct sunlight and backlight conditions on sunny days, which affected the image quality, especially the tapped area, which caused the shape and texture characteristics of the tapped area to be fuzzy and the color of the tapped area to be similar to the background, resulting in the failure of the new tapping line location. The tapped area in the natural rubber tree images that had been cut for one year was difficult to detect as a small target, and the segmentation effect was poor, resulting in the failure to locate the new tapping line. In future research, we will further study the segmentation of the tapped area and the location method of the new tapping line when the natural rubber tree is under direct sunlight on sunny days, backlight on sunny days, and the tapped area has been cut for one year, and explore the method of locating the new tapping line by detecting the key points on the edge of the tapped area.

## 4 Conclusion and future work

To realize the autonomous operation of the rubber tapping robot in the rubber garden environment, this paper proposes a method based on the improved Mask RCNN to detect the tapped area and locate the new tapping line for natural rubber trees. By improving the backbone network, structural parameters, and mask branch of the Mask RCNN, combined with edge detection and logic operation, the detection and segmentation of the tapped area of natural rubber trees and the fine segmentation of the existing tapping line were realized. Finally, the position of the new tapping line was calculated, providing technical support for the automatic natural rubber tapping machine. The specific conclusions are as follows:

(1) Compared with MR(50), IMRB(XA50), IMRA(XA50+FC), MR(101), and IMRB(XA101) network models, IMRA(XA101+FC) had the highest AP and IOU values. Its AP_0.5_, AP_0.75_, AP_0.5-0.95_, and IOU values were 99.6%, 97.62%, 80.59%, and 93.71%, respectively. Compared with MR(101), the AP_0.5_, AP_0.75_, AP_0.5-0.95_, and IOU values of IMRA(XA101+FC) increased by 2.02%, 6.14%, 4.81%, and 4.62%, respectively, indicating that compared with ResNet50, ResNet101, and ResNeXt50 fused with the attention mechanism, ResNeXt101 fused with the attention mechanism had a stronger ability to extract features, and the object detection and segmentation performance of the model had been significantly improved. IMRA(XA101+FC), which changed the anchor box ratio and added a tiny FC layer branch to the mask branch, paid more attention to the target itself, increased information diversity, realized feature enhancement, had the best object detection performance, and improved the segmentation performance of the model.

(2) Compared with Mask RCNN, YOLACT, Cascade Mask RCNN, PointRend, Swin-B Cascade Mask RCNN, FCN, and DeepLabv3, the improved Mask RCNN model proposed in this study had better detection and segmentation performance. The detection accuracy, segmentation accuracy, detection average precision, segmentation average precision, and IOU values of the improved Mask RCNN were 98.23%, 99.52%, 99.6%, 99.78%, and 93.71%, respectively. In addition, under different shooting conditions, the F1 and IOU values of the improved Mask RCNN were higher than those of the Mask RCNN, indicating that, compared with the Mask RCNN, the improved Mask RCNN could better detect and segment the tapped area of natural rubber trees.

(3) The location results of 560 new tapping lines under different shooting conditions showed that the location success rate of new tapping lines on cloudy days was the highest, at 92.11%, which was 1.57% and 3.18% higher than that on direct sunlight on sunny days and backlight on sunny days, respectively. The location success rates of the new tapping line of natural rubber trees where the tapped area had been cut for two and three years were higher than those of the tapped area that had been cut for one year. Under different shooting conditions, the average success rate of the new tapping line location was 90%, the average location time was 0.189 s, the maximum values of the location errors in the *x* and *y* directions were 5.5 and 7 pixels, respectively, and the maximum value of the total location error was 8.2 pixels, which met the location accuracy and speed requirements of the natural rubber tree tapping machine.

At present, the method proposed in this paper can accurately detect the tapped area of natural rubber trees, but the network model is slightly larger, and the segmentation accuracy needs to be further improved. In future research, we will collect images of natural rubber trees of different varieties, expand the dataset of natural rubber trees under different conditions, and study methods to further simplify the network structure and improve the segmentation accuracy. For the location of the new tapping line of natural rubber trees, there is a situation of location failure. The reason for this is that the segmentation effect of the tapped area is poor. In future research, further research will be conducted on the segmentation of the tapped area and the localization of the new tapping line for natural rubber trees, and the method of locating the new tapping line by detecting the key points on the edge of the tapped area will be explored.

## Data availability statement

The original contributions presented in the study are included in the article/supplementary material. Further inquiries can be directed to the corresponding author.

## Author contributions

YC: conceptualization, methodology, software, investigation, and writing – original draft. HZ: investigation and writing – review and editing. JL: writing – review and editing. ZZ: writing – review and editing. XZ: conceptualization, writing – review and editing, project administration and funding acquisition. All authors contributed to this article and approved the submitted version.
